# Molecular interaction of human acetylcholinesterase with *trans*-tephrostachin and derivatives for Alzheimer's disease

**DOI:** 10.1016/j.heliyon.2020.e04930

**Published:** 2020-09-14

**Authors:** Arjun Pitchai, Rajesh Kannan Rajaretinam, Rajasekar Mani, Nagasundaram Nagarajan

**Affiliations:** a Neuroscience Lab, Centre for Molecular and Nanomedical Sciences, Centre for Nanoscience and Nanotechnology (CNSNT), School of Bio and Chemical Engineering, Sathyabama Institute of Science and Technology, Jeppiaar Nagar, Rajiv Gandhi Salai, Chennai, 600119, Tamil Nadu, India; b School of Humanities, Nanyang Technological University, 14 Nanyang Dr, Singapore, 637332, Singapore

**Keywords:** Acetylcholinesterase (AChE), Flavonoids, *Trans*-Tephrostachin derivatives, Enzyme inhibition kinetics, Molecular docking and dynamics, Biochemistry, Neuroscience, Pharmacology

## Abstract

Alzheimer's disease (AD), a neurodegenerative disorder affects more than 35 million people globally. Acetylcholinesterase suppression is the common approach to enhance the well-being of AD patients by increasing the duration of acetylcholine in the cholinergic synapses. Generally, herbal secondary metabolites are reported to be a major resource for acetylcholinesterase inhibitors (AChEIs). *Trans*-tephrostachin was reported from *Tephrosia purpurea* for AChE inhibition. Here, we report on the design, synthesis, and assessment of human acetylcholinesterase inhibitory activity from *trans*-tephrostachin derivatives or analogs as anti-AD agents. The five newly synthesized compounds 4a. 4b, 4c, 4d and 4e displayed potent inhibitory activities with IC_50_ values of 35.0, 35.6, 10.6, 10.3, and 28.1 μM respectively. AChE enzyme kinetic study was performed for the five derived compounds using the Ellman's method. The Lineweaver-Burk and the secondary plots revealed the mixed inhibition for 4a, 4c and 4d whereas 4b and 4e demonstrated competitive inhibition. Molecular docking and molecular dynamics simulations showed the derivatives or analogs of *trans*-tephrostachin attained a high binding affinity and efficacy than the standard drug. In conclusion, *trans*-tephrostachin and its derivative compounds could become effective agents for further drug development to treat AD.

## 1 Introduction

Alzheimer's disease (AD) is a major neurodegenerative disease and cause serious cognitive impariment in human beings. The incremental transition, disorientation and oblivious behaviour in the society are the significant observation in the AD patients. The symptomatic phase of the disease is increasing gradually by memory loss and cognitive impaiment within a period of five years or more [1]. The etiology of AD is not fully known, but certain characteristics, such as low levels of acetylcholine, β-amyloid (Aβ) deposits, aggregation of τ proteins, oxidative stress, inflammation, and biometaldyshomeostasis, are considered to play an important role in the pathogenesis of this disease [2, 3]. Many studies have concentrated on the cholinergic system of the basal forebrain, since AD symptoms are connected with impaired cholinergic function [4]. The loss of cholinergic activity in the brain by cognitive reduction are linked with ageing and AD [5]. Therefore, medicines which can suppress AChE and potentiate central cholinergic activity will be an increasing focus for AD patients [6].

AChE inhibitors (AChEIs) can inhibit the activity of AChE by competitive mechanism through interaction with the catalytic center (CC) of enzyme by binding to the peripheral anionic site (PAS) or by double binding inhibition of AChE [6]. It was reported that the treatment with AChEIs showed symptomatic improvement for a short period [7]. However, the AChE interacts with Aβ through a near-PAS hydrophobic environment to promote the formation of Aβ fibrils [8, 9]. In addition, AChE-Aβ complex increase the neurotoxicity due to Aβ [10]. The noncholinergic trait of AChE in relation to its Aβ-related PAS is an attractive target developed for the design of new anti-dementia drugs [8, 9, 10]. AChE peripheral or dual site inhibitors can reduce the cognitive impairment in AD patients by stopping the Aβ assembling to prolong the neurodegenerative process [11].

According to the cholinergic hypothesis, acetylcholinesterase (AChE) antagonists including galantamine, tacrine, rivastigmine and donepezil are commonly used in clinical practice for AD care [12]. Several medicinal chemists adopted this approach for the development of new compounds to inhibit AChE [13, 14, 15, 16]. There is a vital need to find new and efficient anti-AD drugs to enhance the survival rates of AD patients. Natural products played a major role in drug discovery and production due to their diverse range of bioactivities, minimal toxicity and lesser side effects [17, 18, 19, 20, 21].

Over the past few decades, more studies showed that flavonoids have significant effects on neurological disorders, such as neuro-protective effects [22] AChE inhibitory activity [23], Aβ fibril formation inhibitory activity [24]. Flavonoids have been reported to be attractive natural products that are widely available in nature and have strong biological activity. We have identified AChE inhibitory flavonoid *trans*-tephrostachin from the herbal *Tephrosia purpurea* [25, 26, 27]. Therefore, the compounds of *trans*-tephrostachin and derivatives were designed and synthesized, for anti-AD drug development. Flavonoid scaffold is linked with terminal amine groups through carbon spacers of varying lengths and based on the geometry of AChE to build the dual binding site of AChEIs. The terminal amine groups could inhabit the CAS through cation-p interaction, while flavonoid scaffolds could interact with PAS of AChE through aromatic stacking interactions and thereby protonated at physiological pH. The narrow middle gorge was covered with a flexible carbon spacer and the size of the carbon spacer was modified with the intention of achieving optional validation that could communicate with both AChE's CAS and PAS. A series of *trans*-tephrostachin derivatives with specific basic functional groups (bromoethene, benzyl, nitobenzyl and acetyl) have been designed, synthesized and evaluated for their inhibition of cholinesterases (ChEs). The flavonoids were synthesized using a versatile tool in organic chemistry by Heck reaction (palladium-catalyzed arylation) [28]. There are very few reports were found and our research group initiated a systematic survey on the use of palladium-catalyzed cross-coupling reactions of flavonoids. Iodination of flavonoids and subjected to Heck reactions to give the expected alkenes in moderate to good yields [29, 30]. It was proposed, based on previous considerations, that the integration of various derivatives into a flavonoid scaffold could be an effective strategy for the search for new flavonoid derivatives with possible anti-AD action. Hence in this study, we focused on the chemical synthesis of *trans*-tephrostachin and derivatives. They were evaluated for *in vitro* AChE inhibition assay and *in silico* studies.

## 2 Materials and methods

### 2.1 Chemicals

Chrysin, Iodochloride (ICl) and Palladium (II) acetate, 2-methylbut-3-en-2-ol were purchased from Sigma-Aldrich Chemicals Pvt. Ltd, USA. Triethylamine (TEA) and dimethylformamide (DMF) solvents were obtained from SRL, India with high purity. Column chromatography was performed on silica gel 60 (100–200 mesh). ^1^H & ^13^C NMR spectra were recorded on Bruker DRX 500 in deuterated chloroform (CDCl_3_) and deuterated dimethyl sulfoxide (DMSO-d6).

### 2.2 General method for the synthesis of *trans*-tephrostachin and its derivatives (4a-e)

The commercially available chrysin 1 has been used as a starting substance for *trans*-tephrostatin and derivative synthesis. Firstly, the treatment of chrysin **1** with acid chlorides and potassium carbonate (K_2_CO_3_) provided compound **2**. The iodination reaction of **2** with ICl and acetic acid in DMSO provided **3** [31]. The solution **3** was allowed to Heck reaction to form *trans*-tephrostachin (**4a**) and its other derivatives (**4b-e**) [32,33]. A mixture of compound **3**, triethylamine, and palladium (II) acetate in 15 mL of DMF was heated to 160 °C under nitrogen or argon. The disappearance of starting material was noted. Subsequently the reaction was quenched with water, extracted with chloroform and evaporated. The dried powder was washed using hexane and the end product was further purified by chromatography.

#### 2.2.1 Physicochemical and spectral data for (*E*)-8-(3-hydroxy-3-methylbut-1-en-1-yl)-5,7-dimethoxy-2-phenyl-4*H*-chromen-4-one(4a)

The **4a** was obtained by the 5,7-dimethoxy-8-iodochrysin (**3a**, 408 g, 1 mmol), 2-methylbut-3-en-2-ol (0.86 g), triethylamine (390 mL, 2.798 mmol), and palladium (II)acetate (0.28 g,) in DMF (15 mL) was heated at 160 °C under nitrogen. The final product as a yellow powder with the yield of 79%. ^1^H NMR (500 MHz, CDCl_3_): *δ* 1.26 (t, 6H, *J* = 4.5 Hz,-CH_3_), 2.98 (s, 1H, - OH), 4.00 (s, 6H, -OMe). 6.42 (s, 1H, Ar–H), 6.55 (s, 1H, Ar–H), 6.73 (s, 1H, Ar–H), 6.82 (s, 1H, Ar–H), 7.55 (q, 3H, *J* = 7.2 Hz, Ar–H), 7. 88 (d, 1H, *J* = 7.2 Hz, -Ar-H), 8.02 (t, 1H, *J* = 7.5Hz, Ar–H). ^13^C NMR (125 MHz CDCl_3_): *δ* 29.67, 57.25, 70.26, 90.31, 96.01, 104.88, 105.53, 126.11, 126.47, 128.96, 130.90, 132.48, 158.22, 161.39, 163.29, 164.60, 181.39. Anal. calcd for C_22_H_22_O_5_: C, 72.12; H, 6.05. Found: C, 72.14; H, 6.06.

#### 2.2.2 Physicochemical and spectral data for (*E*)-5,7-diethoxy-8-(3-hydroxy-3-methylbut-1-en-1-yl)-2-phenyl-4*H*-chromen-4-one(4b)

The **4b** was obtained by the **3b** (408 g, 1 mmol), 2-methylbut-3-en-2-ol (0.86 g), triethylamine (390 mL, 2.798 mmol), and palladium (II)acetate (0.28 g,) in DMF (15 mL) were heated at 160 °C under nitrogen. The final product as a yellow powder with an yield of 68%; ^1^H NMR (500MHz, DMSO-d_6_):*δ* 1.24 (s, 6 H, *J* = 4.5 Hz, –CH_3_), 1.43 (t, 6H, *J* = 4.5 Hz, –CH_3_), 1.56 (s, 1H, - OH), 4.24 (q, 4H, *J* = 5.2 Hz, - CH_2_). 6.64 (s, 2H, Ar–H), 7.17 (s, 2H, Ar–H), 7.65 (q, 3H, *J* = 7.2 Hz, Ar–H), 8.23 (d, 2H, *J* = 7.5 Hz, Ar–H).^13^C NMR (125 MHz, DMSO-d_6_): *δ* 14.24, 21.83, 29.99, 32.52, 61.78, 65.26, 91.04,93.87, 96.35, 104.84, 106.13, 107.97, 126.24, 126.49, 128.72, 130.63, 131.57, 155.77, 163.00, 164.27, 181.92. Anal. calcd for C_24_H_26_O_5_: C, C, 73.08; H, 6.64. Found: C, 73.06; H, 6.62.

#### 2.2.3 Physicochemical and spectral data for (*E*)-8-(3-hydroxy-3-methylbut-1-en-1-yl)-4-oxo-2-phenyl-4*H*-chromene-5,7-diyl dibenzoate(4c)

The **4c** was obtained by the (**3c**, 408 g, 1 mmol), 2-methylbut-3-en-2-ol (0.86 g), triethylamine (390 mL, 2.798 mmol), and palladium (II)acetate (0.28 g,) in DMF (15 mL) were heated at 160 °C under nitrogen. The final product as a yellow powder with a yield of 72%; ^1^H NMR (500 MHz, DMSO-*d*_*6*_): *δ* 1.24 (s, 6H, *J* = 4.5 Hz –CH_3_), 2.12 (s, 1H, - OH), 6.45 (s, 3H, Ar–H), 6.73 (s, 3H, Ar–H), 7.12 (d, 4H, *J* = 7.2 Hz, Ar–H), 7.64 (d, 5H, *J* = 7.5 Hz, Ar–H), 8.12 (t, 4H, *J* = 8.2 Hz, Ar–H). ^13^C NMR (125 MHz, DMSO-*d*_*6*_): *δ* 13.97, 14.60, 30.33, 32.17, 36.91, 63.32, 65.53, 105.39, 111.53, 124.89, 126.33, 126.50, 128.91, 129.30, 131.83, 132.16, 163.36, 182.56, 193.88. Anal. calcd for C_34_H_26_O_7_: C, 74.71; H, 4.79. Found: C, 74.73; H, 4.76.

#### 2.2.4 Physicochemical and spectral data for (*E*)-8-(3-hydroxy-3-methylbut-1-en-1-yl)-4-oxo-2-phenyl-4*H*-chromene-5,7-diyl bis(4-nitrobenzoate) (4d)

The **4d** was obtained by the (**3d**, 408 g, 1 mmol), 2-methylbut-3-en-2-ol (0.86 g), triethylamine (390 mL, 2.798 mmol), and palladium (II)acetate (0.28 g,) in DMF (15 mL) were heated at 160 °C under nitrogen. The final product as a yellow powder with a yield of 58%; ^1^H NMR (500 MHz, CDCl_3_): *δ* 1.27 (s, 6H, –CH_3_), 2.09 (s, 1H, –OH), 6.53 (d, 2H, *J* = 7.2 Hz, -Ar-H), 6.53 (d, 2H, *J* = 7.2 Hz, Ar–H), 6.73 (s, 1H, Ar–H), 6.99 (s, 3H, Ar–H), 7.08 (s, 3H, Ar–H), 7. 62 (t, 3H, *J* = 7.5 Hz, -Ar-H), 8.02 (t, 3H, *J* = 7.2 Hz, Ar–H). ^13^C NMR (125 MHz DMSO-*d*_*6*_):*δ* 29.69, 98.89, 105.77, 106.13, 125.32, 127.53, 128.43, 131.90, 138.21, 138.85, 149.48, 155.14, 183.20. Anal. calcd for C_34_H_24_N_2_O_11_: C, 64.15; H, 3.80; N, 4.40. Found: C, 64.17; H, 3.82; N, 4.42.

#### 2.2.5 Physicochemical and spectral data for (*E*)-8-(3-hydroxy-3-methylbut-1-en-1-yl)-4-oxo-2-phenyl-4*H*-chromene-5,7-diyl diacetate (4e)

The **4**e was obtained by the (**3e**, 408 g, 1 mmol), 2-methylbut-3-en-2-ol (0.86 g), triethylamine (390 mL, 2.798 mmol), and palladium (II)acetate (0.28 g,) in DMF (15 mL) were heated at 160 °C under nitrogen. The final product as a yellow powder with a yield of 67%; ^1^H NMR (500 MHz, CDCl_3_): *δ* 1.27 (s, 6H, –CH_3_), 1.45 (s, 3H, –OCH_3_), 1.50 (s, 3H, –OCH_3_), 2.45 (s, 1H, - OH), 6.55 (t, 1H, *J* = 6.5 Hz -Ar-H), 6.81 (s, 1H, Ar–H), 6.87 (s, 2H, Ar–H), 7.59 (t, 3H, *J* = 7.5 Hz, Ar–H), 7.86 (d, 1H, *J* = 7.2 Hz, -Ar-H), 8.04 (d, 1H, *J* = 7.2 Hz, Ar–H). ^13^C NMR (125 MHz, DMSO-*d*_*6*_): *δ* 21.49, 30.34, 69.00, 71.23, 106.13, 108.59, 114.61, 127.53, 129.06, 129.99, 132.83, 143.87, 144.46, 155.78, 156.78, 161.45, 164.29, 165.19, 166.18, 168.05, 181.91. Anal. calcd for C_24_H_22_O_7_: C, 68.24; H, 5.25. Found: C, 68.26; H, 5.27.

### 2.3 Enzyme inhibition assays

#### 2.3.1 Determination of the inhibitory effects on *h*AChE activities

Human acetycholinesterase (*h*AChE) activities were determined with a slight modification of Ellman's colorimetric protocol [37]. The synthesized *trans*-tephrostachin and derivatives (4a to 4e) were soluble in DMSO and diluted in phosphate buffer. The bioactivity was determined by evaluating the hydrolysis rate of acetylthiocholine iodide (ATCI, 0.1 mM) with the final volume of 300 μL with a phosphate buffer of 180 μL of 100 mM, pH 7.3 and 2 mM DTNB 0.09U of *h*AChE (Sigma) were applied to this solution and pre-incubated for 45 min at room temperature. The 96-well microplate was read in a microplate reader (Ensight Multimode Reader, Perkin Elmer) at 412 nm after adding DTNB and ATCI. The enzyme kinetic parameters were determined by incubating the substrate at multiple concentrations with or without test inhibitor by double-reciprocal (Lineweaver- Burk) plots in 0.09U of *h*AChE. From the Lineweaver-Burk plots, the inhibition mechanisms were measured graphically. The kinetics were performed using a series of concentrations of 4a to 4e and substrate AChI at 412 nm. The quantitative datas were expressed as mean ± SD results from three independent experiments. GraphPad Prism version 6.01 (GraphPad Software Inc., San Diego, USA) was used for regression analysis to calculate the IC_50_ values and the enzyme kinetic parameters (Km, Vmax). The IC_50_ and enzyme kinetics values (Vmax and Km) were calculated by this formula IC_50_ Y = min + max-min/1+(X/EC50)^−Hillslops.^ . Vmax and Km Y = Vmax∗X/(Km + X).

### 2.4 Molecular docking methodology

Molecular docking process follow the lock and key formula by the receptor and ligand binding in a perfect orientation. Generally, in molecular docking program, ligands are always kept flexible and bind with receptor in different possible conformations. It may undergo conformation changes when ligand interact to the receptor by following the “Induced fit” model procedure. Molecular docking for the receptor and ligands docking were performed with Auto Dock Vina [34]. The polar hydrogen and further additional charges were added on the ligand by utilizing the Auto Dock tools. Grid map and size on the ligand were defined through grid map [35]. To perform molecular docking analysis lamarckian genetic algorithm was applied. With different conformation level, individual docking analysis were repeated for ten times and computerized to stop the run after completing 250,000 energy evaluations. The molecular docking evaluations were included in the translational step with 0.2 Å and with a population size of 150. The molecular docking output were prioritized based on the frequency of the most possible ligand binding site and free energy score.

### 2.5 Molecular dynamics simulations methodology

GROMACS 2018.3 [36] package have been used for molecular dynamics simulation run. The three dimensional molecular structures of acetylcholinesterase complex with *trans*-tephrostachin derivatives were used as the initial material for MD simulation run. To minimize the molecular energy, GROMOS53a6 force field was used and then solvated in a 0.9 nm size cubic box by setting periodic boundary conditions with the simple point charge water molecule. PRODRG server was used to generate the GROMACS compatible topology files for ligands. After neutralizing the complexes, the systems were subjected for energy minimization by adopting the steepest descent energy minimization procedure. A position restrained dynamics simulations (NPT and NVT) have been implemented to equilibrate the system with the thermal condition at 300 K for 300 ps. The equilibrated systems were then subjected to MD simulation run for 50 ns with a pressure of 1 atm and a constant temperature of 300 K. The integration time step was set to 2 fs. The non-bonded cutoff was set based on the atomic threshold value of 8 Å. Interatomic potential energies were calculated based on the Lennard-Jones mathematical formula with cutoff threshold value of 0.9 nm. Long-range electrostatic interactions were calculated by appling particle mesh ewald algorithm. The Lincs algorithm was used to measure the hydrogen bond length in the simulated systems [36]. The trajectory snapshots were stored for each pico-seconds for structural analysis. The RMSD, minimum distance and the hydrogen bonds, were calculated using the Gromacs utilities g_rms, g_mindist and g_hbond.

## 3 Results and discussion

### 3.1 Synthesis and characterization of *trans*-tephrostachin derivatives

**Scheme 1:**
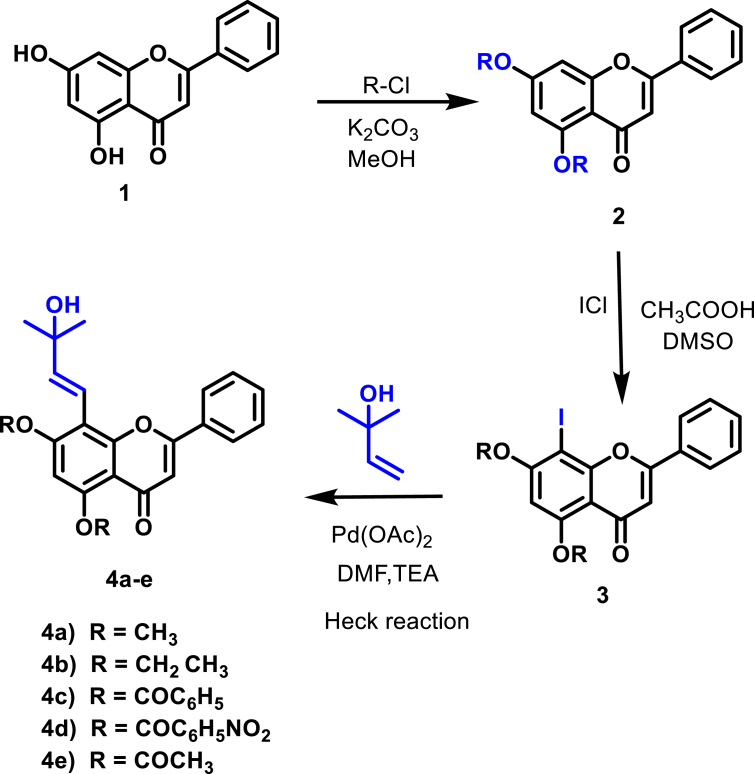
Synthesis of *trans*-tephrostachin and derivatives (4a-e).

Chrysin derivatives (**3a-e**) reacted with 2-methylbut-3-en-2-ol in the existence of palladium (II) acetate and TEA as a catalyst in DMF solvent, resulted in 58–79% yield of *trans*-tephrostachin and its derivatives **(4a-e**) as shown in Scheme 1. Structure of reaction time and product yields are given in Table 1. The ^1^H NMR spectra of *trans*-tephrostachin derivatives (**4a-e**) showed the methyl proton appeared in the range of 1.0–1.5 ppm and aromatic proton at 6.00–8.5 ppm. However ^13^C NMR studies showed peaks at 17–41 ppm, 110–162 ppm and 169–200 ppm relevant to the alkyl carbons, aromatic carbons and carbonyl group respectively.

**Table 1:** Structure of reaction time and product yields.

No	R	t (h)	Yield (%)
1	**4a**	8	79
2	**4b**	7	68
3	**4c**	8	72
4	**4d**	8	58
5	**4e**	8	67

### 3.2 Biological activities

#### 3.2.1 Determination of the inhibitory effects on *h*AChE activities

**Figure 1:**
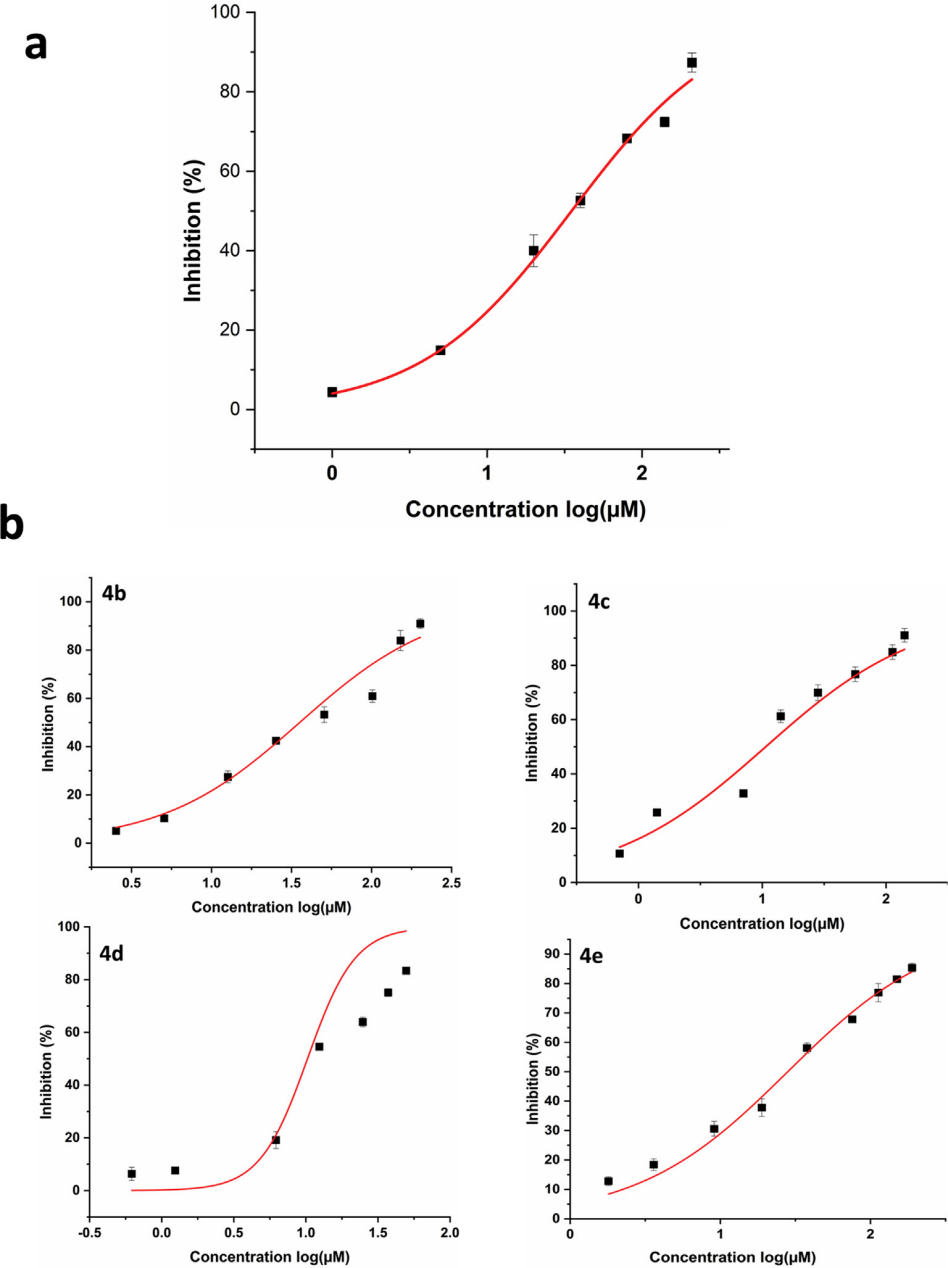
Inhibition of human AChE: 1a (4a) synthesized *trans*-tephrostachin. 1b (4b to 4e) derivatives. 50% inhibition was observed at 35.0 ± 1.2μM, 35.6 ± 2.8 μM, 10.6 ± 2.8 μM, 10.3 ± 2.2 μM and 28.1 ± 2.1 μM respectively.

**Figure 2:**
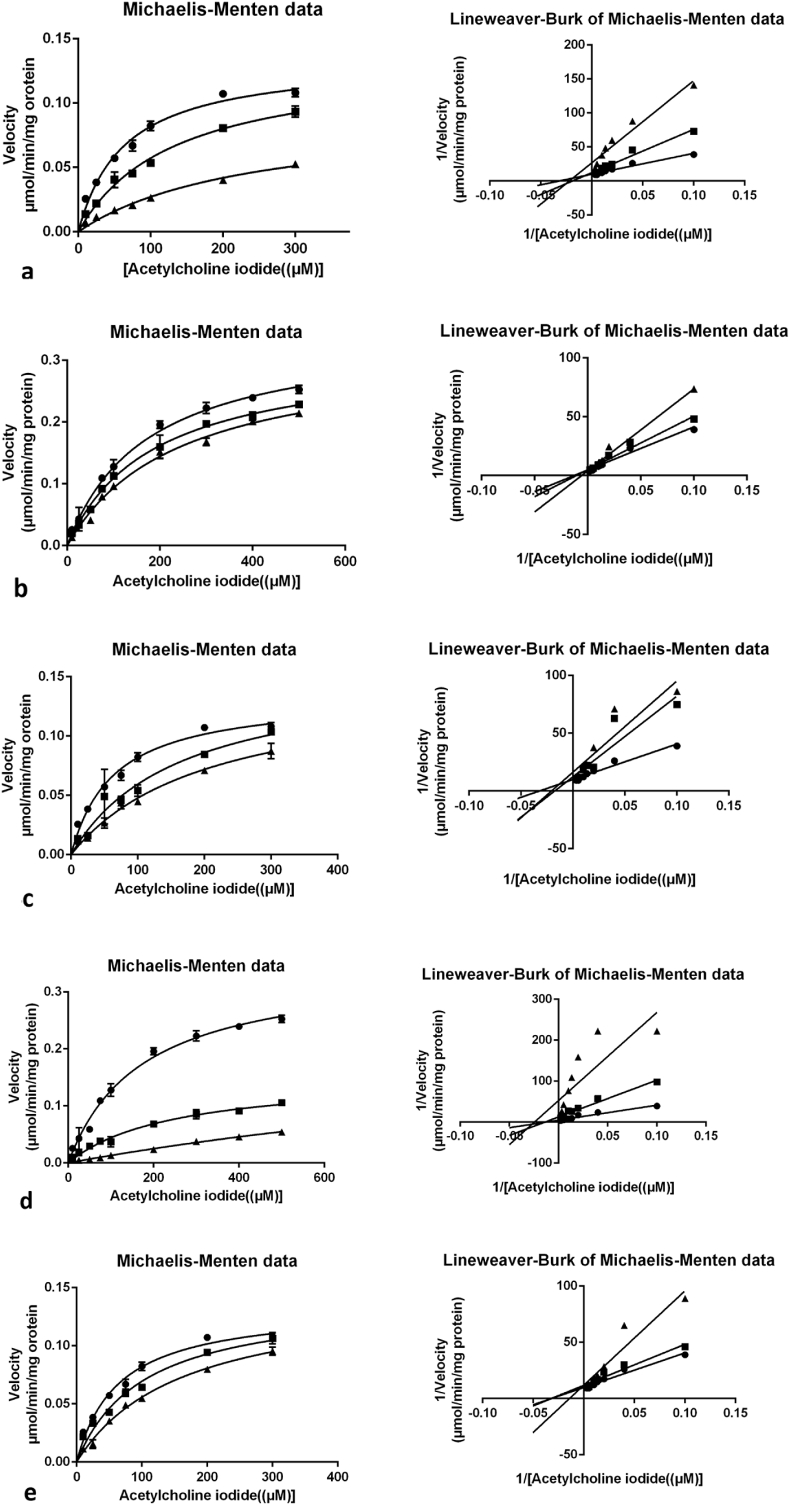
Michaelis Menten and Lineweaver–Burk plots of cholinesterase inhibition by synthesized *trans*-tephrostachin (4a) and derivatives (4b – 4e).

AChE inhibitory activity has been tested on synthesized *trans*-tephrostachin (4a) and derivatives (4b-4e). Donepezil has been used as a positive control drug to allow the comparison of results. An evaluation of the therapeutic potential in the treatment of AD of target compounds 4a, 4b, 4c, 4d and 4e were measured for AChE inhibitory activity using the Ellman method [37]. To find the inhibitory effects, the test compounds were incubated with multiple concentrations of human AChE. All the experimental compounds were showed significant AChE inhibitory activity. . The IC_50_ of *h*AChE with synthesized compounds 4a-4e were found to be 35.0 ± 1.2 μM, 35.6 ± 2.8 μM, 10.6 ± 2.8 μM, 10.3 ± 2.2 μM and 28.1 ± 2.1 μM respectively (Figure 1 and Table 2). These compounds were designed from the *trans*-tephrostachin. It was isolated and structuraly characterized from *Tephrosia purpurea* in our previous studies [25, 26, 27]. To investigate the mechanism of *trans*-tephrostachin derivatives against AChE, kinetic studies were conducted for all compounds. The Lineweaver-Burk plots and the corresponding secondary plot of compounds 4a - 4e are shown in Figure 2. Such plots presented a secondary plot of Km/Vmax versus test compound concentrations for the Km and Vmax. Table 3 shows the Km and Vmax values of synthesized compounds and the Ki of human AChE for 4a-4e is 29.3 ± 7.4 μM, 206.3 ± 12.0 μM, 46.4 ± 9.5 μM, 8.254 ± 1.7 μM and 197.62 ± 10.0 μM respectively. The kinetic study of Lineweaver-Burk for 4a, 4c and 4d demonstrated a decrease in Km and Vmax values. 4b and 4e exhibited an increase in Km value without affecting the maximum velocity of enzyme activity. Compound 4a is a *trans*-tephrostachin which was isolated and structurally characterized previously [25, 26]. The synthesized *trans*-tephrostachin showed the similar mode of inhibition kinetics with IC_50_ value and correlated with the isolated *trans*-tephrostachin from *T. purpurea* [25]. Therefore, the results showed AChE inhibition was found to be a mixed type inhibition for 4a, 4c and 4d while 4b and 4e showed competitive inhibition. The enzyme inhibition mechanisms showed mixed type for 4a, 4c and 4d which showed very comparable with donepezil inhibitory mechanisms, while the 4b and 4e showed very similar inhibition mechanisms of galantamine [25]. Thus the above results revealed that the five synthesized compounds are having two different molecular interaction results, which gives further research interest to understand its molecular actions in neurodegenerative conditions.

**Table 2:** 50% inhibition concentration of AChE inhibitor.The datas are the average of three sets of assays performed.

S.No	Compound Name	IC_50_ Concentration (μM)
1	4a	35.0 ± 1.2
2	4b	35.6 ± 2.8
3	4c	10.6 ± 2.8
4	4d	10.25 ± 2.2
5	4e	28.1 ± 2.1

**Table 3:** Inhibitory effect of synthesized *trans*-tephrostachin 4a and derivatives 4b – 4e.

Compound	V_max_	Km (μM)	Ki (μM)	Inhibition type
Without Inhibitor	0.34 ± 0.01	173.5 ± 17.8	_____	_____
**4a**	0.13 ± 0.08	234.3.0 ± 35.0	29.3 ± 7.2	Mixed inhibition
**4b**	0.34 ± 0.01	240 ± 23.6	206.3 ± 12	Competitive inhibition
**4c**	0.14 ± 0.1	205.5 ± 34.7	46.42 ± 9.5	Mixed inhibition
**4d**	0.15 ± 0.01	247.6 ± 36.7	8.3 ± 1.7	Mixed inhibition
**4e**	0.36 ± 0.2	265.2 ± 37.18	197.62 ± 10.1	Competitive inhibition

IC_50_ values for human AChE of compounds 4a-4e indicated moderate binding affinities. All synthesized compounds exhibited the potential inhibitory activity against *h*AChE. Some molecules of flavonoid-derivatives have observed with mixed and competitive nature of inhibition [38, 39] against AChE such as hesperetin derivatives, prenylated xanthones [40], in comparison to normal dehydroevodiamine (IC_50_ 37.8 μM), quercetin and tiliroside showed great inhibitory effect with IC_50_ values of 19.8 and 23.5 μM respectively. At the same time 3-methoxy quercetin and quercitrin demonstrated less activity with IC_50_ value of 37.9 and 66.9 μM, respectively [41]. At present, acetylcholinesterase inhibitors are the only accepted therapy for treating AD. Since a significant amount of facts suggests that the oxidative stress is actively involved in neurodegenerative diseases. The major source of novel natural therapeutic agents against AD has been greatly extended to flavonoids [42]. Hence, AChE inhibitory flavonoids can be appealing sources of AD care. Our results have established the anti-AChE activity of the five compounds that are consistent with previous research. This is the first acetylcholinesterase study involving inhibitory activity of the 4a-4e compounds to the best of our knowledge.

### 3.3 Molecular binding affinity

**Figure 3:**
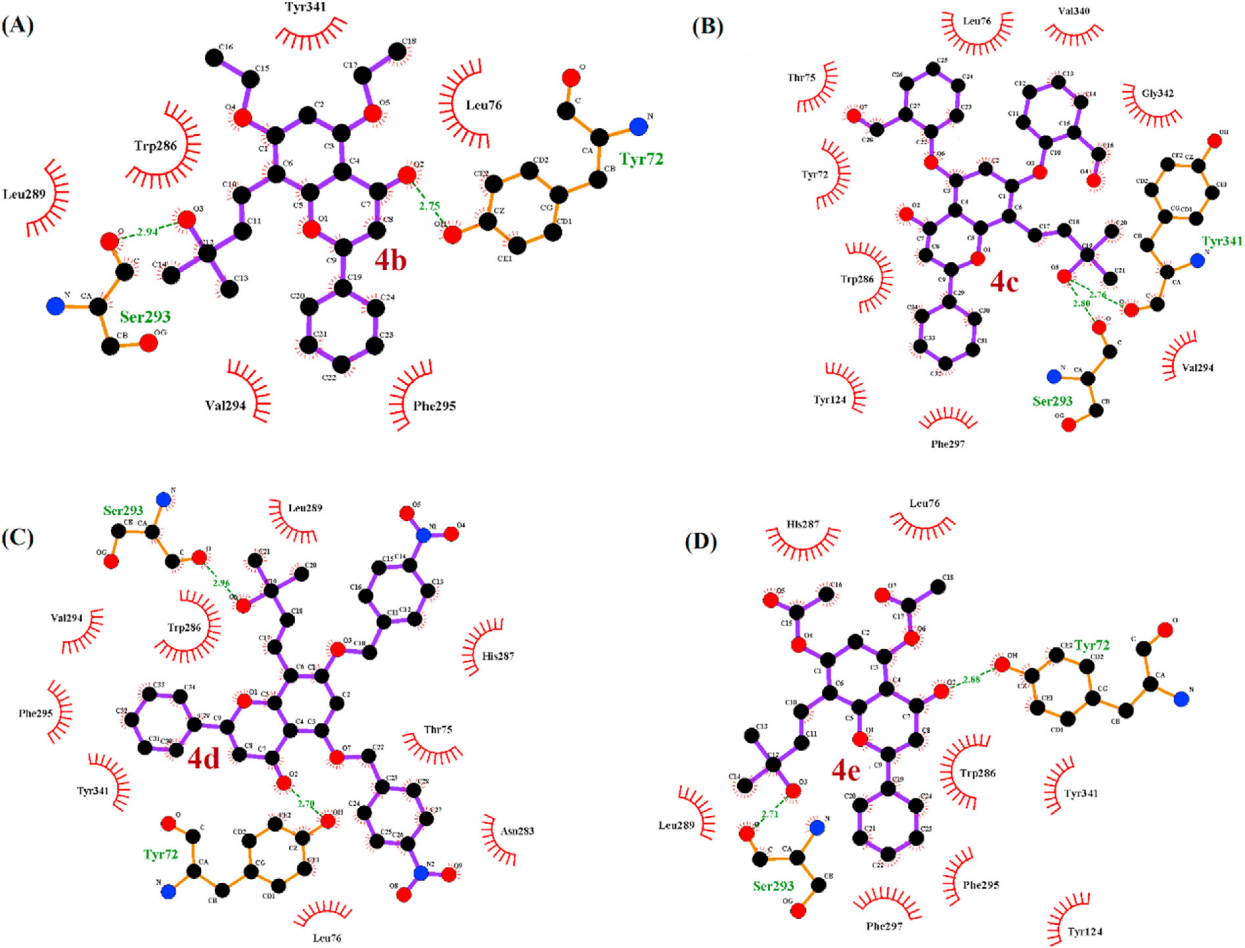
Ligplot analysis of *trans*-tephrostachin analogues interaction with human acetylcholinesterase enzymes (AChE). (A) Analogue 4b interaction with AChE and form two hydrogen bonds with residues TYR72 and SER293. (B) Analogue 4c interaction with AChE and form two hydrogen bonds with residues SER293 AND TYR341. (C) Analogue 4d interaction with AChE and form two hydrogen bonds with residues TYR72 and SER293. (D) Analogue 4e interaction with AChE and form two hydrogen bonds with residues TYR72 and SER293.

Molecular docking studies were carried out to understand the affinity between AChE and *trans*-tephrostachin derivatives. Initial 3D structure of human acetylcoline esterase retrieved from RCSB PDB 3D molecular data bank which was resolved at 2.90 Å assigned with PDB ID 4PQE. Details of the drug interacting amino acid residues were obtained from the resolved AChE complex crystal structures with donepezil and galantamine. They were deposited with ID 4EY7 and 4EY6 in the PDB database, respectively. The donepezil binding residues were identified as W86, E202, S203, G120, G121, G122, F295, F297, Y337 and H447. Galantamine binding residues were identified as W86, E202, S203, F295, W286, Y341, Y337 and H447. Structure based precision docking were conducted by setting grid on the active site of AChE - donopezil and AChE - galantamine interaction mode. It was further noted that the four derivatives of *trans*-tephrostachin were bound on the standard drug interacting site with AChE (Figure 3). *Trans*-Tephrostachin binds strongly with AChE residues D74, W86, N87, S203, G120, G121, Y124, G126, F295, F297, Y341 and F388 with a binding energy value of -7.3 Kcal/mol [25]. Similarly, the derivative or analog 4b binds in the ATP binding pocket by interacting with the amino acids Y72, L76,S293, V294, F295, L289, W286 and Y341 by having the binding energy value of -7.2 Kcal/mol. 4b form two H-bonds with AChE residue Y72 and S293 (Figure 3A). The derivative 4c binds in the ATP binding pocket and interacted with amino acid H287, T75, N283, L76, Y72, Y341, F295, V294, S293, W286 and L289 with a binding energy of -6.9 Kcal/mol. 4c form two H-bonds with AChE residue Y293 and Y341 (Figure 3B). The derivative 4d binds in the ATP binding pocket and interacted with amino acids Y72, L76, Y124, W286, L289, S293, F295, F297, H287 and Y341 with binding energy value of -7.2 Kcal/mol. 4d form two H-bonds with AChE residue Y72 and S293 (Figure 3C). The derivative 4e binds in the ATP binding pocket and interacted with amino acid Y72, W75, L76, Y124, V340, W286, S293, V294, F297, G342 and Y341 with the binding energy value of -7.3 Kcal/mol. 4e form two H-bonds with AChE residue Y72 and S293 respectively (Figure 3D).

### 3.4 Molecular dynamics simulations

**Figure 4:**
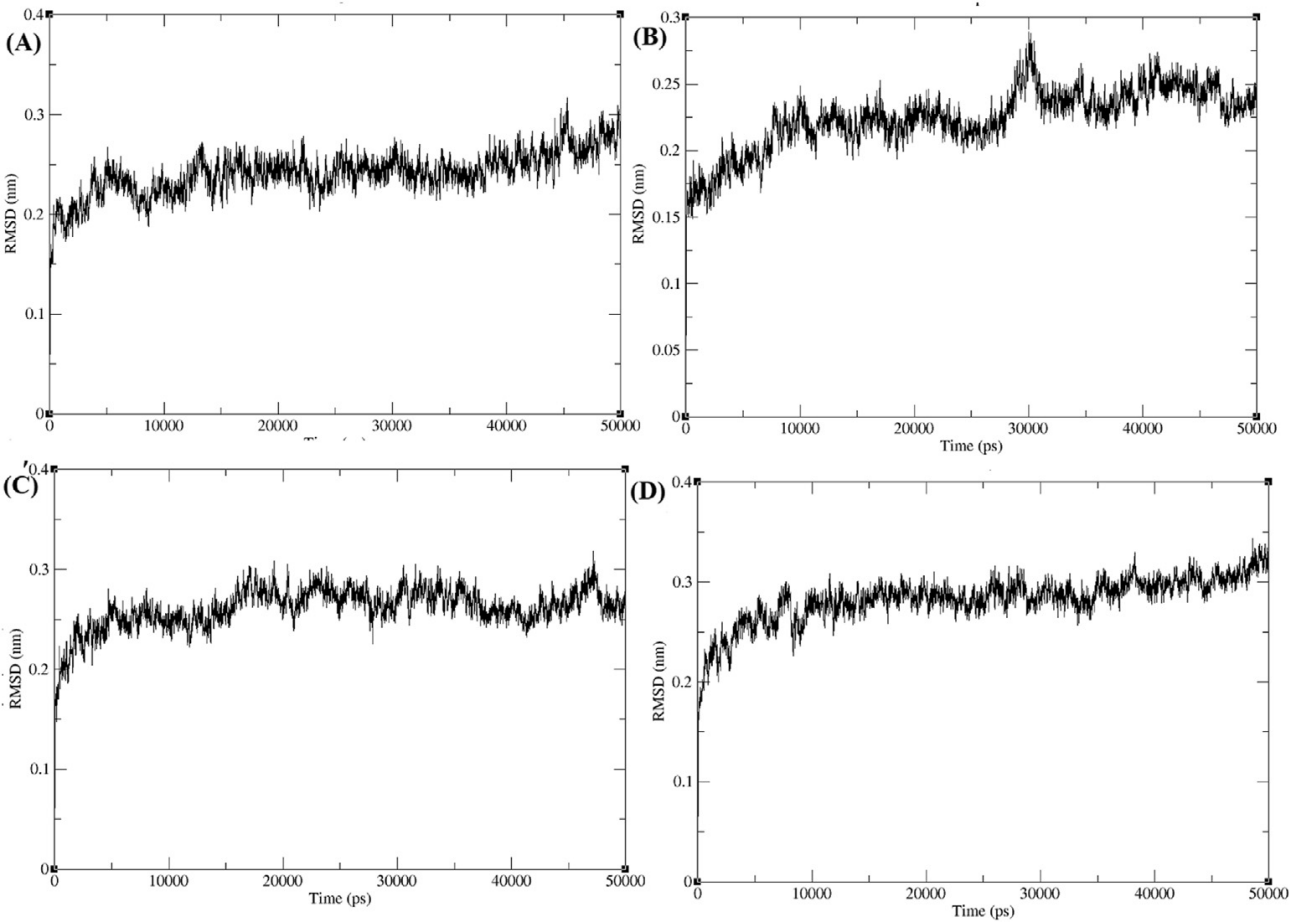
Root mean square deviation (RMSD) of acetylcholinesterase enzymes (AChE) in complex with *trans*-tephrostachin analogues. (A)RMSD of AChE-4b complex (B) RMSD of AChE-4c complex (C) RMSD of AChE-4d complex (D) RMSD of AChE-4e complex.

A 50 ns molecular dynamics simulations were carried out to explore the binding efficacy of docked AChE-4b, AChE-4c, AChE-4d and AChE-4e complexes. The MD trajectory have been analyzed to calculate the backbone RMSD obtained during simulation period. The RMSD graph analysis (Figure 4) shows that AChE-4b, AChE-4c, AChE-4d and AChE-4e complex obtained the equilibrium phase at~5,000 ps and fluctuated in the range ~0.25 Å. All four derivatives were obtained stable equilibrium during the simulation period. However, it was reported in our previous studies that the standard drugs donepezil and galantamine in complex with AChE obtained the equilibrium at ~25,000 ps in which the conformations fluctuated around ~3.25 Å and ~0.3 Å respectively [25]. In comparision with standard drugs, *trans*-tephrostachin derivatives obtained less RMSD values which indicates high binding efficacy.

**Figure 5:**
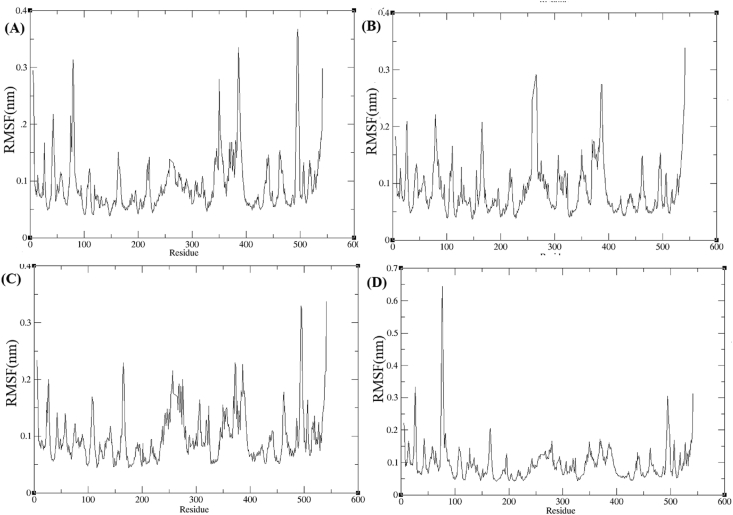
Root mean square fluctuation (RMSF) of acetylcholinesterase enzymes (AChE) in complex with *trans* -tephrostachin analogues. (A) RMSF of AChE-4b complex (B) RMSF of AChE-4c complex (C) RMSF of AChE-4d complex (D) RMSF of AChE-4e complex.

C-alpha RMSF analysis was performed to calculate the flexibility of AChE residues in complex with *trans*-tephrostachin derivatives 4b, 4c, 4d and 4e. RMSF analysis showed AChE in complex with *trans*-tephrostachin derivatives (Figure 5). In comparing with the RMSF results for AChE-donepezil and AChE-galantamine [25] the average range of fluctuation were noted less for AChE-*trans*-tephrostachin derivatives. This showed higher stability of AChE in complex with *trans*-tephrostachin derivatives. From RMSF analysis it is elucidated that derivatives of *trans*-tephrostachin obtained high binding efficacy with AChE in compared with standard drugs.

**Figure 6:**
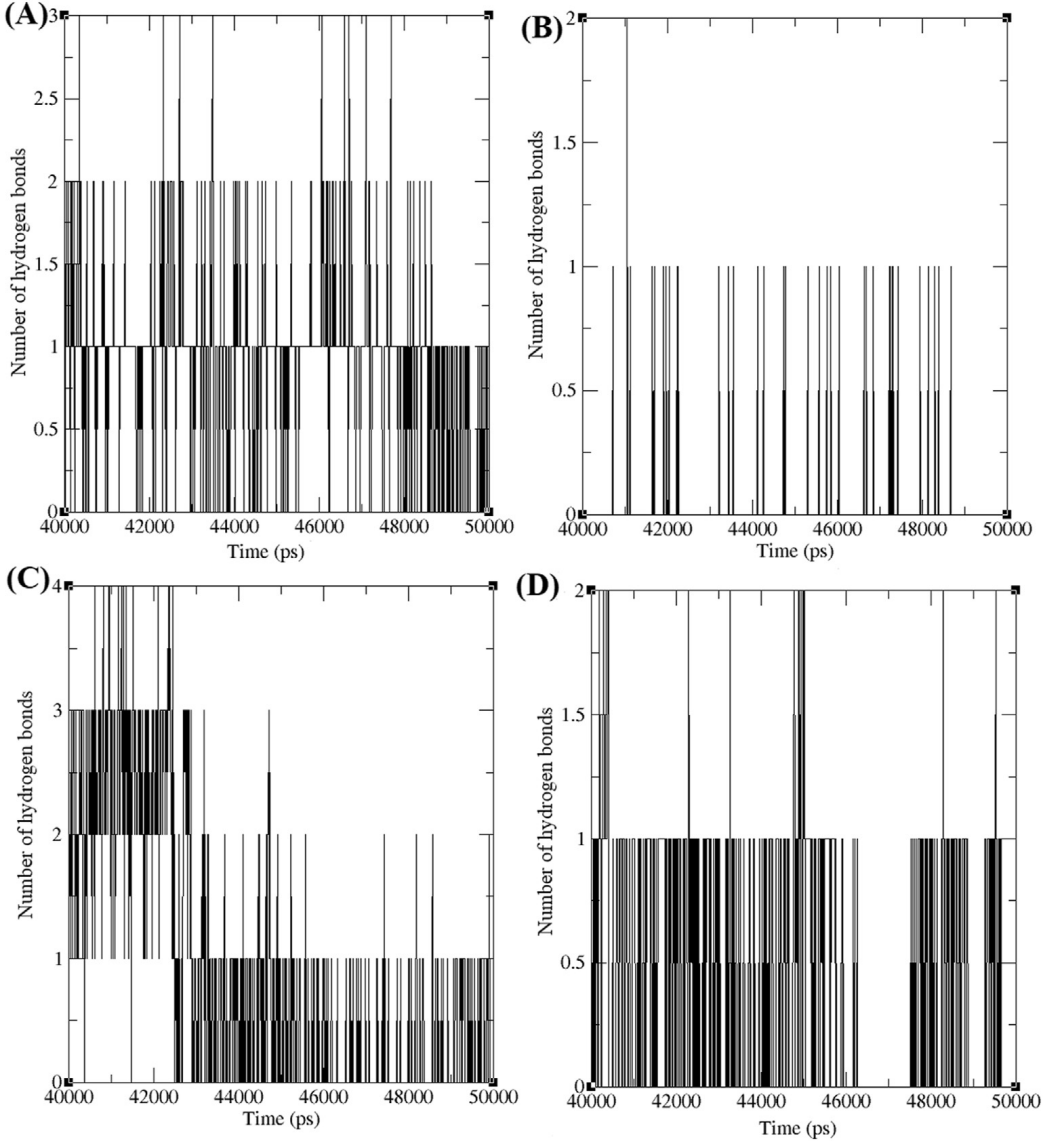
Number of hydrogens formed between acetylcholinesterase enzymes (AChE) and *trans* -tephrostachin analogues in the last 10ns of simulation period. (A) Hydrogen bond formed between AChE-4b complex (B) Hydrogen bond formed between AChE-4c complex (C) Hydrogen bond formed between AChE-4d complex (D) Hydrogen bond formed between AChE-4e complex.

A geometrical equation was setup to identify the hydrogen bonds (H-bond) present between AChE-*trans*-tephrostachin derivatives complexes. The existence of hydrogen bonds are determined by the bond orientation and distance between atoms of both molecules. The bond angle is smaller than 60.0° and the distance between acceptor A and donor D is shorter than 3.5 Å that can be assumed as hydrogen bonds. For instance, the atomic distance between X…A and H…A will be considered as very strong bond which occur in the range of 2.2–2.5(Å) and 1.2–1.5(Å) respectively and weak bond are considered in the range of 3.0–4.0(Å) and 2.0–3.0 (Å) respectively [25]. Individual hydrogen bonds are weak and easily broken, but many hydrogen bonds together can be very strong. The number of H-bonds between AChE-4b, AChE-4c, AChE-4d and AChE-4e were calculated from the trajectory value of last 10 ns (Figure 6). AChE-4b, AChE-4c, AChE-4d and AChE-4e were maintained 1–3, 1–2, 1–4 and 1-2 H-bond. However our previous study reported that the AChE-donepezil and AChE-galantamine were obtained one H-bond in the last 10 ns of simulation period [25]. Hydrogen bond analysis elucidated the better binding efficacy of *trans*-tephrostachin derivatives with AChE, as they obtained high number of H-bonds.

**Figure 7:**
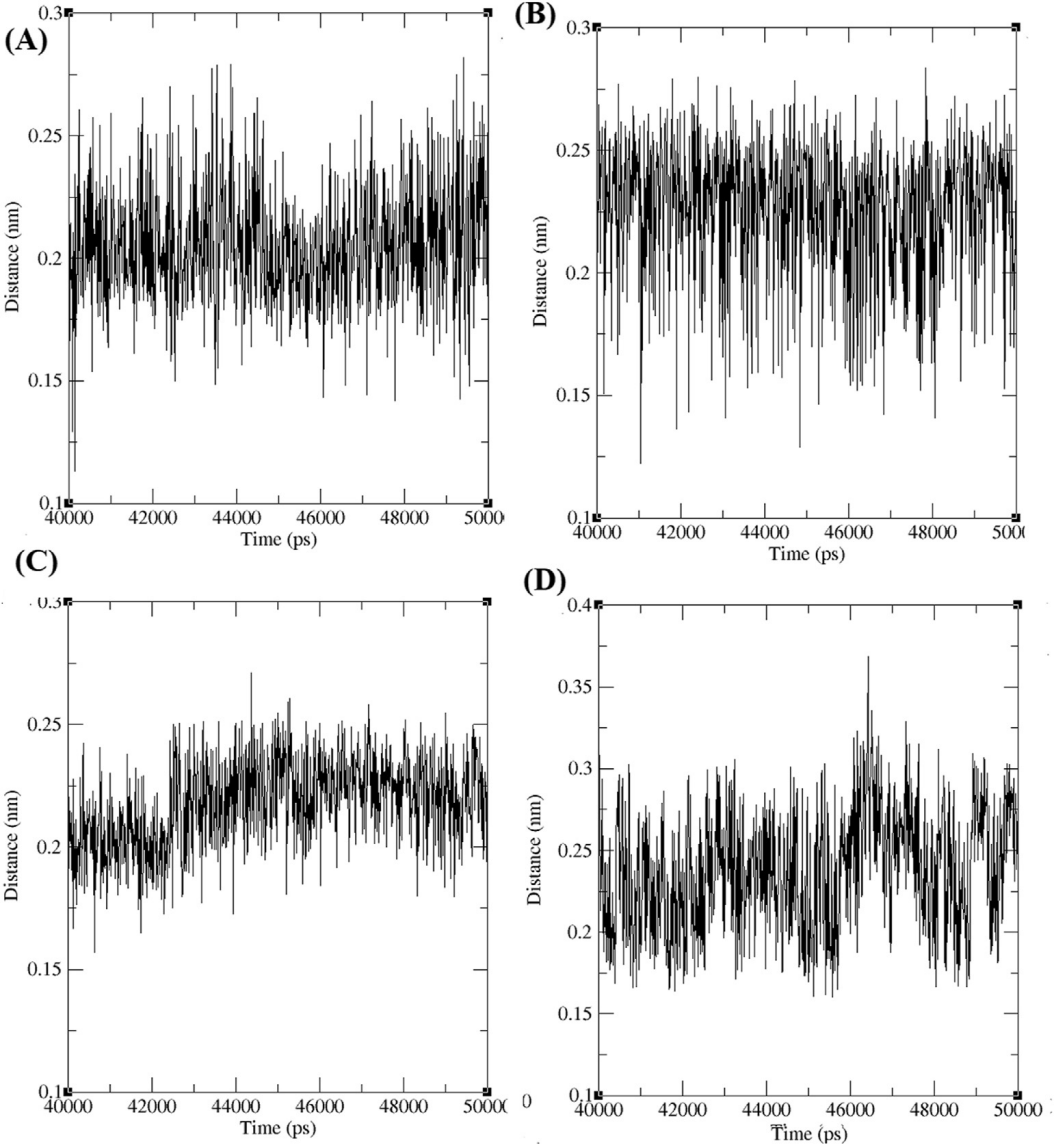
Minimum distance formed between acetylcholinesterase enzymes (AChE) and *trans*-tephrostachin analogues during the last 10ns of simulation period (A) Minimum distance between AChE-4b complex (B) Minimum distance between AChE-4c complex (C) Minimum distance formed AChE-4d complex (D) Minimum distance between AChE-4e complex.

The minimum distance between AChE-4b, AChE-4c, AChE-4d and AchE-4e were noted as ~0.12 to ~ 0.27 nm, ~0.12 to ~0.27 nm, ~0.16 to ~0.26 and ~0.16 to ~0.35 nm respectively (Figure 7). Lesser minimum distance is observed between AChE-*trans*-tephrostachin derivatives than the AChE-donepezil and AChE-galantamine complexes [25]. It indicates the tight binding between molecules resulted in better stability for AChE-*trans*-tephrostachin derivatives than AChE-galantamine and AChE-donepezil.

**Figure 8:**
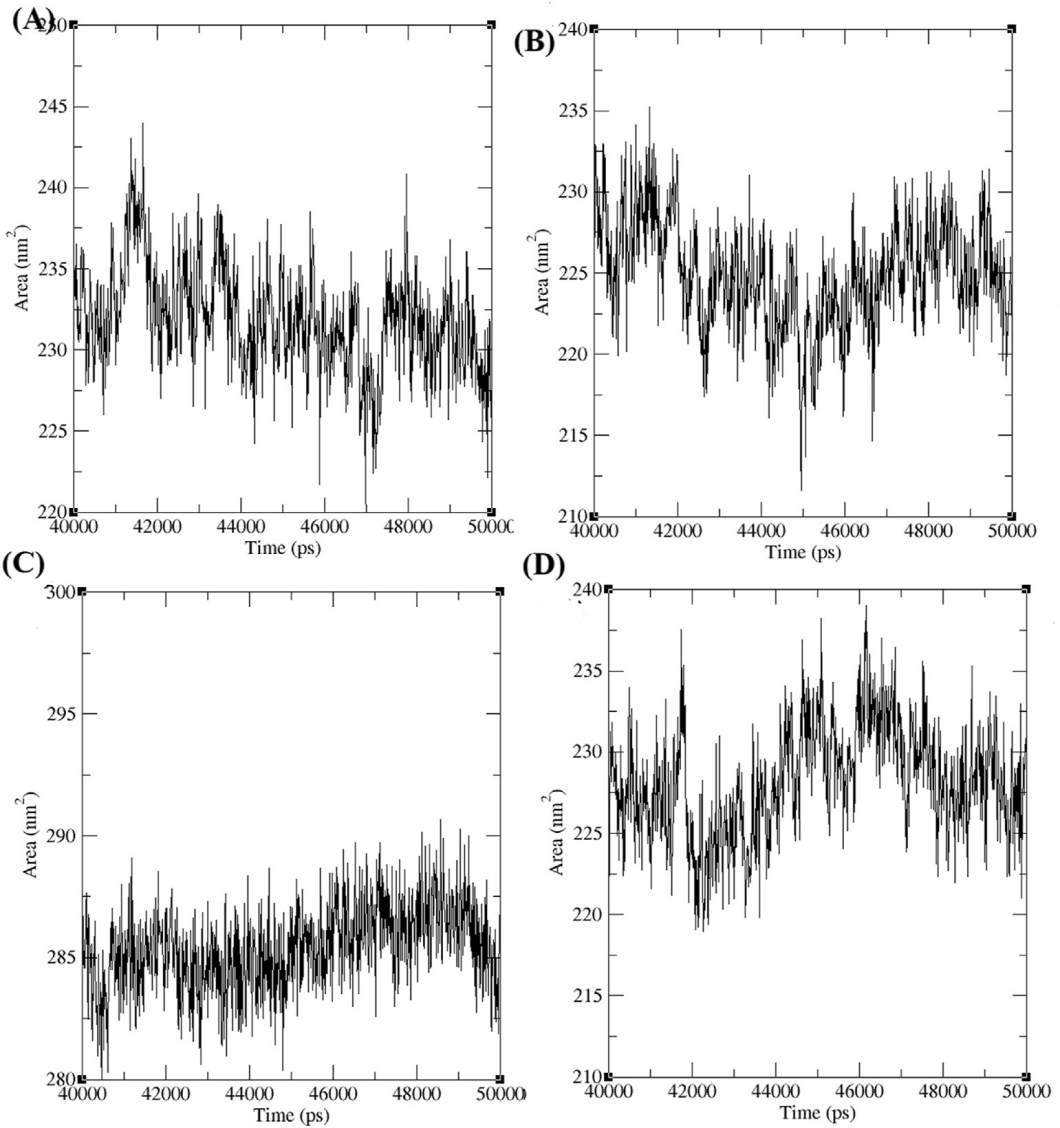
Solvent accessible surface area (SASA) of acetylcholinesterase enzymes (AChE) in complex with *trans*-tephrostachin analogues. (A) SASA of AChE-4b complex (B) SASA of AChE-4c complex (C) SASA of AChE-4d complex (D) SASA of AChE-4e complex.

Solvent accessible surface area (SASA) is measured as molecular surface exposed to the solvent molecule. The SASA was mesured for AChE in complex with 4b, 4c, 4d, and 4e. The SASA was calculated for the AChE-4b, AChE-4c, AChE-4d and AChE-4e complex and it was observed around~220 nm^2^ to ~245 nm^2^, ~212 nm^2^ to ~235 nm^2^, ~280 nm^2^ to ~290 nm^2^ and ~218 nm^2^ to ~238 nm^2^ respectively from the last 10 ns trajectory value analysis (Figure 8). It has been reported in our previous study ~192 nm^2^ to ~208 nm^2^ and ~190 nm^2^ to ~205 nm^2^ SASA were observed for AChE-donepezil and AChE-galantamine complexes respectively [25]. High SASA was observed for AChE-derivatives (4b-4e) than the AChE-donepezil and AChE-galantamine complexes. The binding of *trans*-tephrostachin analogues in the active site of AChE measured high SASA in comparison with donepezil and galantamine, which elucidated the binding of *trans*-tephrostachin analogues induced less conformational changes in the AChE than the donepezil and galantamine. Further clinical studies are warranted to understand the therapeutic efficay of *trans*-tephrostachin and analogues.

## 4 Conclusion

In summary, *trans*-tephrostachin derivatives were synthesized and tested for their inhibitory activity of acetylcholinesterase. These molucules revealed to be a moderate to good inhibition activity against human acetylcholinesterase enzymes with IC_50_ value from micro molar to sub micro molar range. Molecular docking analysis elucidated the high binding affinity of *trans*-tephrostachin derivatives compared with standard drugs which obtained high binding energy and strong hydrogen bonds. Molecular dynamics simulation analysis also showed the high binding efficacy of the derivatives compared with standard drugs. As evidenced from the results, the synthesized compounds may be considered as a potential anti-AD agent for further developments. Further preclinical and clinical studies of the selected flavonoids should be tested as a lead for further neurotherapeutic potential.

## Declarations

### Author contribution statement

Pitchai Arjun: Conceived and designed the experiments; Performed the experiments; Analyzed and interpreted the data; Wrote the paper.

Rajaretinam Rajesh Kannan: Conceived and designed the experiments; Wrote the paper.

Rajasekar Mani: Performed the experiments; Wrote the paper.

Nagasundaram Nagarajan: Performed the experiments; Analyzed and interpreted the data.

### Funding statement

Rajesh Kannan Rajaretinam was supported by 10.13039/501100010803Department of Biotechnology (DBT), 10.13039/501100004541Ministry of Science and Technology, Govt. of India (San.No: BT/PR6765/NNT/28/618/2012) Arjun Pitchai was supported by Council of Scientific and Industrial Research (10.13039/501100001412CSIR) (File number: 09/1205 (0001) 2k18 EMR-I, Dated: 2 May 2018).

### Competing interest statement

The authors declare no conflict of interest.

### Additional information

No additional information is available for this paper.
